# An Address on the Nature of the Science and Art of Medicine, and Their Relations to the Various Important Interests of the People

**Published:** 1880-05

**Authors:** N. S. Davis

**Affiliations:** Chicago, Ill.


CHICAGO MEDICAL

JOURNAL & EXAMINER.

VOL. XL.—MAY, 1880.—NO. 5.

### Original Lectures.

ARTICLE I.

### AN ADDRESS ON THE NATURE OF THE SCIENCE AND ART OF MEDICINE, AND THEIR RELATIONS TO THE VARIOUS IMPORTANT INTERESTS OF THE PEOPLE. Delivered before the citizens of Lincoln and the members of the Illinois State Medical Society, May 21st, 1879. By N. S. DAVIS, M.D., LL.D., Chicago, Ill.

*Fellow Citizens and Brethren of the State Medical Society:*

In compliance with an earnest request from the Committee of Arrangements, I rise to address you, briefly, on a topic of universal interest and great intrinsic importance. I allude to the Science and Art of Medicine and their relations to the important interests of the whole people. The science of medicine is that field of knowledge which is capable of being used for the prevention, alleviation, and cure of diseases; and consequently for the prolongation ot human life. The art of medicine is the direct or personal application of such knowledge, either in the execution of sanitary measures for the prevention of disease, or in the application of remedies at the bed-side of the sick.

The field of knowledge that constitutes the science of medicine does not consist in a fixed system or aggregation of rules based on one or two theoretic principles or dogmas, as is indicated by various words and phrases in popular use; but is made up of items drawn from all the departments of the natural, physical, and mental sciences. Man, the great central object of the physician's study, both in health and disease, is but the highest link in the chain of animate beings, and the study of the structure and functions of his body, constituting human anatomy and physiology, is aided and illuminated at every step, by the facts embraced in the departments of zoology, natural history, chemistry, and physics. As a living being, man, in all stages of his existence, is in intimate relation to, and constantly influenced by the physical elements that surround him.

Therefore we cannot advance one step in the study of the causes of disease and the laws that govern their action, without a knowledge of the earth, the air, the water, the products of vegable and animal growth and decay; or in more technical language, without entering directly into the departments of general science, called geology, meteorology, topography, hydrology, and general chemistry. Materia medica, which embraces a knowledge of the various substances and agencies that can be used for the prevention and treatment of diseases, draws its material alike from the organic and inorganic kingdoms of nature. The domains of mineralogy, chemistry, botany, and psychology, are all laid under contribution for materials of greater or less value, in the prevention, alleviation, and cure of disease. You will not fail to perceive from these few sentences, the truth that medical science does not consist of a few rules derived from certain theoretical dogmas or supposed laws of morbid action, but is rather a vast accumulation of facts and materials gathered from almost every department of human knowledge. They also show two other facts that should be more generally recognized, namely: first, that true medical science is strictly coeval in its origin with that of the various branches of general science; and, second, that its progress and practical application have kept even pace with the progress and development of all other departments of human knowledge. And not only so, but stimulated by the desire for more facts and agencies capable of practical application in their high calling, the ranks of the medical profession have furnished a large proportion of the most active and successful cultivators of all those branches of science with which medicine stands connected. It is often alleged as an opprobrium or objection to our profession, that its science is unstable and its modes and instruments of practice ever changing. The objectors apparently forget that the same allegation could be made, with equal force, against all the natural or physical sciences, the changes and progress in which, have been so great and rapid as to change the whole aspect of civilization during the last hundred years. That which appears to the uninitiated as instability in our profession, is only the necessary result of progress, and will continue so long as the botanist finds a new plant or shrub; the chemist a new element or combination of elements; the microscopist a new germ, or the professor of physics a new application of the laws of mind or matter. Indeed, it is this progress, *pari passu*, with the advancement in other departments of science, that constitutes the chief glory of modern medicine, and distinguishes it from all the pathies and isms or so-called special schools of medicine both in ancient and modern times. The art of medicine, consisting of efforts to heal the wounded and cure the sick, existed long before there was anything connected with it meriting the name of science. It was in those early days, that so-called schools or special systems of medicine, had their origin. The men, who from official position or superior mental endowments, gained an influence over their fellows, would often invent some theory of disease, accompanied by arbitrary rules of practice, and these being espoused by their followers, became the foundation of a school or system, taking either the name of their inventor or of some prominent feature of the theory itself. Thus the theories and maxims of Hippocrates, Galen, Celsus, Cullen, Hoffman, Brown, Boerhave, Sydenham, and others, each in turn ruled the minds of their followers and constituted the dominant school or system of medicine for the time. But the progress made in the development and verification of all the sciences during the last century, has placed anatomy and physiology, human and comparative; materia medica and chemistry; therapeutics and pathology, on so broad a basis of ascertained facts, that mere closet speculations and theoretical dogmas have been well nigh driven from the field of legitimate medicine. The medical investigator of to-day does not spend his time in idle dreams over the question whether all the ills that afflict humanity arise from one or more imaginary humors; whether they all consist in either primary irritation or in primary debility; or whether they are all subject to one universal law of cure; but he recognizes the all important fact that man, the central object of his study, is simply a living, sensitive being, constantly influenced for good or evil, by all the physical and mental conditions that surround him.

He recognizes the equally important fact, that disease, instead of being an entity, a misterious something pervading the human system, or an equally mysterious infliction of the gods, is simply a deviation, in some direction, from the standard of health, in one or more of the functions or structures of the human body. Hence the student and practitioner of rational scientific medicine must take his scalpel, test-glass, microscope, etc., and enter the domain of nature along side of the chemist, the geologist, the botanist, the comparative anatomist, the mathematician, and the philosopher, and learn the causes and nature of diseases by observation and logical induction.

It is the substitution of observed facts in the place of fanciful theories, and the adoption of inductive methods of reasoning in place of closet speculations, that has so rapidly enlarged the boundaries of medical knowledge, and literally given birth, not only to numerous discoveries of the highest importance, but to the whole grand field of sanitary, preventive, or state medicine, on which is founded all those modern sanitary measures that have contributed so much to the health and comfort of the millions who occupy large cities and closely populated districts, and has added materially to the average duration of human life, during the last half century.

Even during the forty-five years that have elapsed since I entered upon the study of medicine, the domain of its science has increased in every direction, taking in on every side entirely new fields, such as organic chemistry, microscopy, histology, physical diagnosis, public hygiene, etc., and its storehouse of observed facts and logical deductions concerning the causes of diseases, the laws and conditions of their propagation, and consequently the means of their prevention; also, the means for attaining certainty in diagnosis, as well as for alleviating pain and curing disease, have more than doubled. I repeat, then, that medical science is not a system or bundle of metaphysical theories invented by some transcendental philosopher, or a school of speculative philosophy, but it is the selection and appropriation of all those facts and principles from every department of human knowledge that can be made subservient to the grand purpose of preventing or alleviating the ills of our race. Neither is the true physician of to-day, the blind dogmatic follower of a master, whose name he meekly wears upon his collar or nails upon his door-post; but he is preëminently a student of nature and of nature's masterpiece of workmanship—man. He freely draws his facts and materials from every possible source, and uses them in accordance with the dictates of his own well disciplined mind. If he detects the cause of a disease, he endeavors to remove or neutralize it. If his patient is burning with fever and restless with excitement, he endeavors to cool the one and reduce the other. If he is exhausted and feeble he endeavors to sustain him. He gives large doses or small ones, or no doses at all, as his own enlightened judgment may dictate, with none but his own conscience to molest or make him afraid. In a word, the rational, scientific physician, selects his remedies from any and every field where they can be found, and applies them for the prevention and cure of disease in accordance with the principles of common sense, guided by a disciplined and enlightened judgment. Consequently he has no need to attach an *ist*, an *ic*, an *ism*, or a *pathy* to his professional title. It is often said, and the same was repeated by the eloquent member of the legal profession who so cordially welcomed the members of this society to the hospitalities of your goodly city, that the science of medicine has progressed far in advance of the art.

If those who make this assertion will take the trouble to look at the long list of instruments by which we are enabled to explore the most hidden recesses of the human body, not excepting the interior of the delicate organ of vision, by which we not only detect the existence and location of disease, but the exact stage of its advancement, and the consequent greater certainty and accuracy in the application of remedies; if they will look at the list of anæsthetics for the relief of pain, the nervous and arterial sedatives for allaying nervous restlessness and controlling the circulation, the mechanical appliances for dressing wounds and injuries, preventing or relieving deformities, and a thousand other things that have been added directly to the daily practice of our art, during the last fifty years, they will not fail to acknowledge that the practice of our art in all its aspects, has profited to the fullest extent by every step in the advancement of its science. But I am dwelling too long upon the nature of the science and art of medicine, and must hasten to inquire, what are their relations to the interests of the whole people? The answer to this inquiry has already been clearly indicated, in a general way, by the preceding remarks. It is desirable, however, that an answer should be given more direct and in detail. In doing so, I shall discuss these relations under three aspects, namely: their relations to the individual and family circle; to the public health, and to the public morals. The desire to obtain relief from suffering is an instinct of our nature. And such are our individual relations to the ever varying physical and mental influences with which we are continually in contact, that sooner or later almost every individual and family seeks the aid of the medical man. Hence it is no exaggeration to say, that, at some time, every individual must come in contact with his physician, and that, too, in a relation the most intimate and important. And there is probably no class of men, of equal number, whose relations to individuals and families, afford them opportunity for doing more good or more harm, for contributing more to individual happiness or misery, than the members of the medical profession. I shall not stop here to arouse your full appreciation of this personal relation of every individual to his physician by calling to your mind the anxiety, not to say impatience, with which you wait for his approaching footsteps, when either accident or disease is holding life in jeopardy or torturing you with pain. Neither will I attempt to describe the painful anxiety with which the mother listens to each word and watches each motion or expression of him into whose care she has committed her sick and tender child. No man or woman in this audience who thinks for one moment, can fail to perceive their own direct personal interest in all that relates to the education, the skill, the intelligence and the integrity of the medical profession. Not one of you know the day or the hour when your dearest earthly interests may not be in the keeping of some one of its members. Is it not, then, your individual duty to lend your influence to all such measures as are calculated to secure to the membess of that profession the highest degree of intelligence, skill, and virtue?

If the relations of the profession are thus interwoven with the most important and sacred interests of every individual and family, they are no less so in regard to everything pertaining to the public health and public prosperity. We are too apt to think of the physician only in his daily work of administering to the sick, and forget that it is the practical application of the facts and principles of the science he has been cultivating from generation to generation, that has led to all the improvements in the construction and ventilation of dwellings and public buildings, the widening of the streets, the sewering of cities and drainage of country districts, the adoption of measures for preventing the contamination of the water supplies; the removal of filth and the prevention of overcrowding, whether in cities, work-shops, schools, public charities, prisons, or on ships; the sanitary management of armies, and the limitation of the spread of contagious and infectious diseases. Yet all this is true. For while the introduction and spread of the Christian religion has modified the asperities and cruel passions of man, awakened all the kindlier and nobler attributes of his nature, and created within him a *desire* to alleviate the sufferings of those around him, it has been, at every step of progress, the practical application of medical science and skill that has guided every measure really promotive of the public health. And yet the benefits capable of being derived from this source are only in the infancy of their development. With the advanced condition of chemistry and other physical sciences, and the improved appliances and methods for the most minute and searching investigations in all directions, it only requires patience and a cordial co-operation between the profession and the public, or its representatives in authority, to achieve results for the public good, more important and enduring than any that now grace the pages of medical history. The public often accuse the profession with being too conservative; too much wedded to old doctrines and usages; too slow and reluctant to assent to new truths and additional facts. Your representative in his excellent address of welcome in our first morning session here, re-produced this charge, and it constitutes the common breastwork behind which the advocates of every preposterous theory, from the blunt expressions of stolid ignorance by Samuel Thompson, to the transcendental vagaries of Hahnemann, constantly take refuge.

If we refuse to stultify the dictates of common sense, and set aside all the ascertained laws of mind and matter, for the purpose of persuading ourselves that the potency of any body or substance increases in mathematical ratio to the attenuation of its atoms, and that the true way to remove the symptoms of any one disease is to give an inconceivably small part of something that is supposed to be capable of producing symptoms the nearest possible in likeness to those we wish to remove, we are at once written down as illiberal and bigoted. In a word, if we do not deny the evidence of our senses and fully believe that the sun's rays increase in their power and influence in direct ratio to their scantiness, and abrogate our common sense by consenting that the best way to remove a public nuisance is by creating another as near like it as possible, we are gravely told that the grand discoveries of Harvey, Jenner, Wells, and others, were opposed by their contempories, and therefore (note the logic) we must be bigoted and illiberal. The truth is, that if we, as a profession, err in this matter, it is certainly not in the direction of conservatism or adherance to old doctrines, but in the too great readiness to adopt novelties—to confound coincidences with causes—and to draw important conclusions from insufficient data, or from an incomplete observation of the facts. And a careful examination of the whole history of our profession will show that every important discovery or improvement hitherto made, has been actually adopted before the disappearance of the generation cotemporary with such event, and just as fast as a just regard for an adequate examination of the nature of the facts on which it rested could be made. It is a natural and most important tendency of the enlightened and disciplined mind, when asked to assent to any given proposition, to demand and carefully scrutinize the facts on which it rests.

It is, indeed, this tendency, that lies at the foundation of all true advancement, in any and every department of human knowledge. Ignorance and want of mental discipline are the prolific parents of credulity and error. It is the duty of the community, therefore, to encourage, and in some directions, afford special aid to the profession in devising and carrying into execution plans for patiently observing and recording facts; pursuing special investigations, for the purpose of deliberately making important practical conclusions. I allude here especially to the enactment and maintenance of well devised laws for enabling the profession to put on record complete statistics of births, deaths, the prevalence of epidemic diseases, the keeping of complete meteorological records, the making of exact topographical surveys, and the institution of carefully devised experimental researches. Many of the questions and problems pertaining to the origin, laws of progress, and consequent means of the prevention of epidemic diseases, in the proper solution of which the highest interests of the people are involved, cannot, from the extent and complex nature of the elements to be considered, be reliably solved without a combination of observers acting in harmony through a series of years. Such combinations, the profession in every part of the country, are not only willing but anxious to make. But to be successful, they need, in some respects, the cordial coöperation, and to some extent, the pecuniary aid of the people through their municipal, State, and perhaps national legislatures.

I am sure that the non-professional part of the audience I now address will give due heed to these suggestions. But I have intimated that our profession bore, in some way, an important relation to the public morals, as well as to the public health.

That every enlightened and influential class of citizens, necessarily exerts more or less influence for good or evil, over the general tone of public morals will be conceded by every thoughtful individual. When we remember, however, that the members of the medical profession have access to every fireside, and become the special advisers in regard to the habits, modes of life, and mental and physical training, as well as the confidential supervisors of the morbid effects of vice and folly, in all classes of human society, it is easily seen that the influence they do or can exert over public morals is paramount and all-pervading, and carrying with it a responsibility seldom fully appreciated, I fear, by the profession itself. When we remember how large a part of all the vices, crimes, and even diseases of every community, are more or less dependent upon the use of alcoholic drinks and the indulgence of licentious propensities, and how great an influence the firm and united action of the profession could exert if brought to bear in all the intercourse of its members with the community, in regard to these indulgencies, we cannot exagerate the importance of their influence or the extent of their responsibility. But time will not permit me to enlarge upon this part of my theme, neither is it necessary before an audience so intelligent as the one before me.

If I have succeeded in conveying even an imperfect idea of the nature of the science and art of medicine, and their important relations to the highest interests of every civilized community, you will certainly agree with me in the proposition that everything which relates to the education, the organization, and the encouragement of the medical profession, is worthy of your most cordial attention. In conclusion, I must address a few words more directly or exclusively to my brethren of the State Medical Society. From an active intermingling with the profession in all its relations for little less than half a century, I may venture to ask your attention to the following suggestions:

First. By watching the proceedings of all our social organizations, I have been strongly impressed with what has seemed to me a great loss in valuable results from the immense labors of our profession, on account of what may be called fragmentary methods of work. Each individual investigates and writes from the single standpoint of observation occupied by himself; when a large proportion of the questions and problems awaiting solution absolutely require coincident observations in many places, by many individuals, on uniform plans, and through several years of time.

And if a part of the time and attention of every Medical Society was devoted to carefully devising such plans of investigation, and a part of its funds devoted to the procuring of the necessary means and instruments for carrying on the work, requiring regular reports of progress every year, we would make far more rapid progress both in the science and art of our profession. The annual results from such well devised lines of continuous observations and records would generally be comprised in a dozen pages of our transactions, but they would be pages of real additions to our stock of knowledge, worth far more than a volume of conflicting and isolated facts. The American Medical Association, at its recent session, appointed a committee to start work in this direction, and I hope this society will cordially coöperate with the general work of that committee.

Second. I wish to urge upon you, and through you upon the whole profession of the State, the great importance of more completely organizing the profession in every county and district, so that the benefits of social intercourse and combined action shall be far more completely realized.

In union and frequent intercourse there is not only strength, but progress, elevation, enjoyment, friendship and purity. Let us then toil on, shoulder to shoulder, in the great work of preventing, alleviating, and curing the diseases of our fellow men, until the simple name of physician shall be so honored that no man will dare to qualify it by any other adjective than the one applied to the apostle Luke in the days of the Son of Man.

